# Furo[3,2-c]coumarin-derived Fe^3+^ Selective Fluorescence Sensor: Synthesis, Fluorescence Study and Application to Water Analysis

**DOI:** 10.1038/s41598-020-63262-7

**Published:** 2020-05-04

**Authors:** Norfatirah Muhamad Sarih, Alexander Ciupa, Stephen Moss, Peter Myers, Anna Grace Slater, Zanariah Abdullah, Hairul Anuar Tajuddin, Simon Maher

**Affiliations:** 1 Department of Electrical Engineering and Electronics, University of Liverpool, Brownlow Hill, Liverpool, L69 GJ UK; 2 Department of Chemistry, Faculty of Science, University of Malaya, 50603 Kuala Lumpur, Malaysia; 3 Materials Innovation Factory, University of Liverpool, 51 Oxford St, Liverpool, L7 3NY UK; 4 Department of Chemistry, University of Liverpool, Crown St, Liverpool, L69 7ZD UK

**Keywords:** Fluorescent probes, Sensors, Fluorescence spectroscopy

## Abstract

Furocoumarin (furo[3,2-c]coumarin) derivatives have been synthesized from single step, high yielding (82–92%) chemistry involving a 4-hydroxycoumarin 4 + 1 cycloaddition reaction. They are characterized by FTIR, ^1^H-NMR, and, for the first time, a comprehensive UV-Vis and fluorescence spectroscopy study has been carried out to determine if these compounds can serve as useful sensors. Based on the fluorescence data, the most promising furocoumarin derivative (2-(cyclohexylamino)-3-phenyl-4H-furo[3,2-c]chromen-4-one, **FH**), exhibits strong fluorescence (ФF = 0.48) with long fluorescence lifetime (5.6 ns) and large Stokes’ shift, suggesting **FH** could be used as a novel fluorescent chemosensor. FH exhibits a highly selective, sensitive and instant turn-off fluorescence response to Fe^3+^ over other metal ions which was attributed to a charge transfer mechanism. Selectivity was demonstrated against 13 other competing metal ions (Na^+^, K^+^, Mg^2+^, Ca^2+^, Mn^2+^, Fe^2+^, Al^3+^, Ni^2+^, Cu^2+^, Zn^2+^, Co^2+^, Pb^2+^ and Ru^3+^) and aqueous compatibility was demonstrated in 10% MeOH-H_2_O solution. The **FH** sensor coordinates Fe^3+^ in a 1:2 stoichiometry with a binding constant, K_a_ = 5.25 × 10^3^ M^−1^. This novel sensor has a limit of detection of 1.93 µM, below that of the US environmental protection agency guidelines (5.37 µM), with a linear dynamic range of ~28 (~2–30 µM) and an R^2^ value of 0.9975. As an exemplar application we demonstrate the potential of this sensor for the rapid measurement of Fe^3+^ in mineral and tap water samples demonstrating the real-world application of FH as a “turn off” fluorescence sensor.

## Introduction

Coumarin is an aromatic heterocyclic compound made up of two fused six-member aromatic rings, between benzene and pyrone, to form as a benzopyrone. The academic literature contains an abundance of information regarding the synthesis and bioactivities of coumarin derivatives^1–3^. Research involving this ring system has been applied to a wide range of areas including pharmaceuticals^4^, optical brighteners^5^, fluorescents^6–14^ and laser dyes^15^. Recently, we developed a novel mixture of simple organic fluorescents, including furocoumarin, to generate high purity white light emission when applied as a coating to a commercial UV LED^16^. Furocoumarins are one of the coumarin derivatives that can be classified into two groups, i. furan fused benzene ring (psoralen and angelicin) and ii. furan fused lactone ring (furo[3,2-c]coumarin, furo[2,3-c]coumarin and furo[3,4-c]coumarins)^17^. Both psoralen and angelicin compounds are commonly studied because of their abundance in nature compared to the fused furan on the lactone ring^17^. In this study, furo[3,2-c]coumarin has been chosen as a suitable fluorescent heterocyclic candidate as it gives an excellent yield based on published reports^18–20^. Furthermore, the synthesis method for furo[3,2-c]coumarin is both efficient and straightforward (one-pot). It is found in natural products, for example, rhizome of Salvia miltiorrhiza Bunge and exhibits potent biological activity (antitumor, antioxidant, anticoagulant, antifungal, anticancer) with several therapeutic applications^21^. Nair and co-workers reported their preparative procedure which involves a [4 + 1] cycloaddition with *in-situ* generated heterocyclic coumarin methides and isocyanides^18^. Since coumarins typically show excellent spectroscopic properties, high stability and low toxicity^22^, we hypothesized that furo[3,2-c]coumarin derivatives could have potential as fluorescent sensor probes.

The study of fluorescent probes for metal ion detection is a vibrant research field, attracting great interest due to both the importance of detecting heavy metals but also because this sensing approach can offer high sensitivity and fast response times with relatively simple instrumentation requirements^23–25^. Due to the low concentrations at which metal ions are present, for example in biosystems and in the environment, high-sensitivity probes are essential for practical applications^26,27^. In recent years, a large number of fluorescent sensors from coumarin derivatives have been reported for metal ion detection^28^, such as Cu^2+^ ^29–32^, Zn^2+^ ^33–37^, Al^3+^ ^38,39^, Mg^2+^ ^40–42^ and Fe^3+^ ^43–47^. Reference^48^ gives an overview of some of the sensing materials used for Fe^3+^ detection.

Among the metal ions, iron is an essential trace element found in living organisms, and both its deficiency and excess are associated with various disorders, such as Alzheimer’s, Parkinson’s disease^49–51^ and anemia^52^. An excessive amount of iron in the human body can cause toxic damage to various organs including the heart and liver^52^, whilst a lack of iron is related to weakened cognitive growth and decreases the capacity for physical work^53^. In severe excess it is known to be lethal and death has occurred following human ingestion of ~40 mg/kg^54^. The major source of daily iron intake for humans is from food (e.g., green vegetables contain 20–150 mg/kg^55^) with drinking water (assuming an average concentration of 0.3 mg/L) accounting for ~0.6 mg of daily intake. Iron concentration in surface waters is usually <~1 mg/L but much higher concentrations are encountered in groundwater (e.g., >50 mg/L). Excess iron in the environment can also arise due to chemical treatment processes (e.g., coagulation) and from corrosion of ferrous materials. In the USA, the environmental protection agency (EPA) guidelines state that the maximum level of Fe^3+^ in drinking water is 5.37 µM^56^, and in the UK, the drinking water inspectorate (DWI) has set a maximum concentration limit for total iron at 200 µg/L^57^.

The analysis of Fe^3+^ is of great importance for various application areas including biomedical^58^, environmental^59^ and aquatic^60^. In previous work successful attempts have been reported for the detection of Fe^3+^ ^43–47^. However, in each case, selectivity is not demonstrated for some heavy metals (that exhibit properties similar to those of Fe^3+^) which could interfere with detection^61^. For example, we note that Ru^3+^, which amongst the variety of transition metal ions, theoretically, has the greatest similarity to Fe^3+^, is not tested for potential interference. Ruthenium is mainly used in the electronics^62–64^ and chemical industries^65,66^, but it also used for biomedical purposes such as anti-cancer drugs^67,68^. Therefore, for any Fe^3+^ fluorescent probe, it is important to extensively demonstrate selectivity, testing with other heavy metals including ruthenium, as it can be present in the environment^69^, biological systems^70^ and water^71^ samples.

Herein, for the first time, we perform a fluorescent study of furo[3,2-c]coumarin derivatives. In particular, we show that the derivative, 2-(cyclohexylamino)-3-phenyl-4H-furo[3,2-c]chromen-4-one (**FH**), is as an effective fluorescent sensor which exhibits high selectivity for Fe^3+^, tested against 13 other competing metal ions, including Ru^3+^ and Fe^2+^. Finally, we demonstrate the potential of this novel chemosensor for the rapid measurement of Fe^3+^ in real water samples.

## Results and discussions

**Figure 1:**
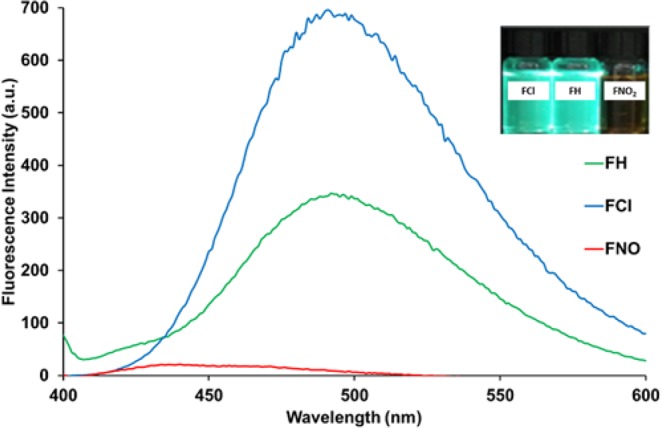
Fluorescence Spectra of Furocoumarin derivatives (**FC**, **FH**, **FNO**_**2**_) in ethanol. Inset: Photograph image of furocoumarin in ethanol under UV lamp illumination.

**Figure 2:**
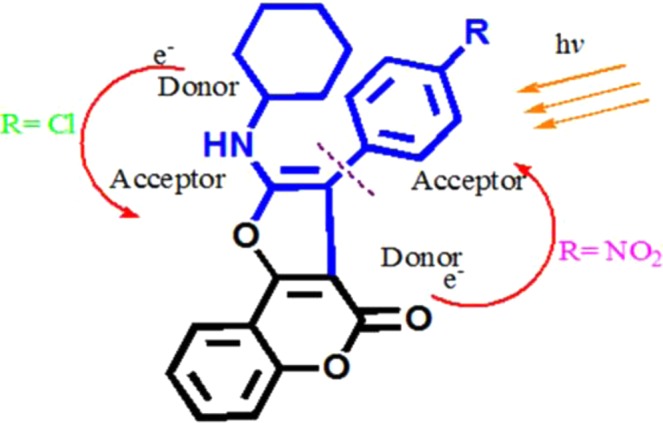
Possible mechanisms whereby chloro- substituent (R = Cl) donates electron through aromatic ring compared with nitro- substituent (R = NO_2_).

The structures of the furocoumarin derivatives (**FH**, **FCl**, and **FNO**_**2**_) were characterized by ^1^H NMR and FTIR. These results are in good agreement with the chemical structures for furocoumarin from the literature^18,19^. Table 1 summarizes the UV-Vis and fluorescence spectroscopy data of **FH**, **FCl** and **FNO**_**2**_. Fig. S1, shows the UV-Vis spectra of **FH**, **FCl** and **FNO**_**2**_ in ethanol. In Fig. 1, the fluorescence spectra of **FH** and **FCl** show higher intensity than **FNO**_**2**_. The main contributing factor responsible for the high fluorescence intensity of furocoumarin is related to its planar and rigid structure^72^. Fluorescence of **FNO**_**2**_ was severely quenched, contrary to the responses for **FH** and **FCl**. Chloro- in **FCl** is a weaker electron withdrawing group (EWG) than -NO_2_ in **FNO**_**2**_, however, the chloro- substituent can also donate through the aromatic ring, which has a high electron density, as the atom is enriched with non-bonding electrons. Therefore, it can be through a π-electron delocalization promoter rather than a nitro group, which acts as a relatively strong EWG as illustrated in Fig. 2. In this case, chlorophenyl would be a donor group to the furocoumarin moiety (an acceptor group). It has been reported that the EWG decreases electron density of the aromatic ring with the exception of the halogen substituent group^73^. The EWG of the nitro group in the benzene ring (nitroaromatic) has empty π^∗^ orbitals of low energy, which are good acceptors of electrons. Therefore electron-rich fluorescent molecules can potentially undergo strong quenching via a photoinduced electron transfer (PET)^74^, fluorescence resonance energy transfer (FRET) or electron exchange energy transfer with nitroaromatics^75–77^. Hence, we attribute the higher fluorescence intensity to the chloro- over the nitro- substituent.

**Table 1:** Concentration [M], Absorbance (Abs), fluorescence lifetimes (τ) and quantum yield (ФF) for fluorescence properties of furocoumarin derivatives in ethanol solution. nd = not determined.

Compounds	[M]	Abs	Molar Abs	λ_ex_ (nm)	λ_em_ (nm)	Ф_F_	Stokes shift (nm)	τ (ns)
**FH**	1.00 × 10^−6^	0.37	2.00 × 10^5^	375	492	0.48	127	5.61
**FCl**	1.00 × 10^−6^	0.20	3.70 × 10^5^	375	491	1.00	126	4.17
**FNO**_2_	1.00 × 10^−5^	0.22	5.70 × 10^4^	380	440	nd	60	nd

### Fluorescence and UV–Vis titration studies of FH with other metal ions

**Figure 3:**
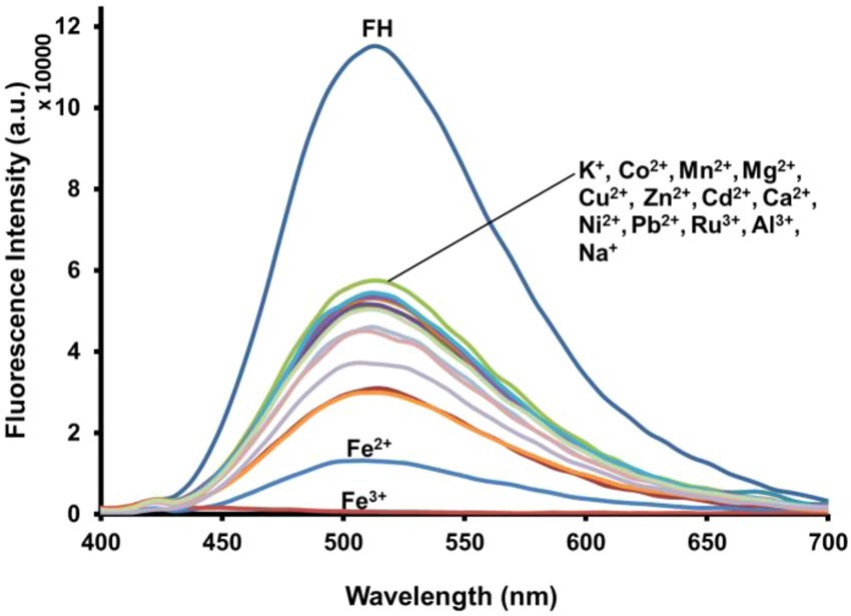
Fluorescence spectra of FH (0.5 μM) in the presence of different metal ions (100 equiv.) in methanol.

The photophysical complexation studies of **FH** with an extensive series of metal salts including: Na^+^, K^+^, Mg^2+^, Ca^2+^, Mn^2+^, Fe^2+^, Fe^3+^, Al^3+^, Ni^2+^, Cu^2+^, Zn^2+^, Co^2+^, Pb^2+^ and Ru^3+^ in methanol, was performed using fluorescence spectroscopy. As shown in Fig. 3, the mixture of **FH** with Fe^3+^ was the only test sample that exhibited no fluorescence emission (i.e., turn-off) in the wavelength range from 430 to 700 nm. Remarkably, in the presence of 50 μM of various metal ions, fluorescence spectra of **FH** exhibited an appreciable fluorescence emission except in the case of Fe^3+^, which resulted in a noticeable turn-off fluorescence response. This fluorescence spectral change was also observed visually when examined with a UV transilluminator (380 nm) as illustrated in Fig. S2. The interaction of **FH** with Fe^3+^ leads to an immediate fluorescence turn-off, while for the other metal ions, a slight fluorescence quenching is observed by the naked eye. As mentioned, the planar and rigid structure of the **FH** molecule makes it a highly fluorescent compound. However, when chelation occurs, there is a transfer of charges within the fluorescent ligand-metal system which then causes fluorescence quenching^78,79^. Therefore, it can be inferred that the fluorescence quenching of **FH** in the presence of Fe^3+^ is due to a ligand-metal charge transfer (LMCT) mechanism. This suggestion is supported by considering the paramagnetic nature of Fe^3+^ with an unfilled d shell, this would take part in the energy and/or electron transfer processes leading to quenching of the fluorescence^80,81^. We suspect, when Fe^3+^ binds with **FH**, the fluorescent opens a non-radiative deactivation channel induced by the unfilled d shell, resulting in fluorescence quenching due to electron transfer^82^. Thus, the mechanism of LMCT could happen promptly due to the strong paramagnetic quenching property of Fe^3+^, leading to a severe fluorescence quenching effect (i.e., turn-off) to coordinate between **FH** and Fe^3+^.

**Figure 4:**
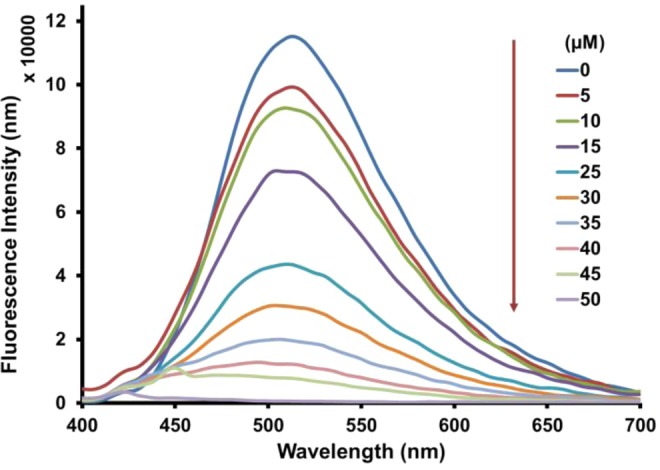
Fluorescence emission spectra of **FH** (0.5 μM) titrated with Fe^3+^ (0–100 equiv.) in methanol.

To gain a quantitative evaluation of the relation between the change in emission intensity of **FH** and the amount of Fe^3+^ interaction, a fluorescence titration experiment was carried out with varying concentrations of Fe^3+^ (Fig. 4). The emission intensity of the peak at 511 nm was systematically quenched by increasing the concentration of Fe^3+^ from 5 to 50 μM. Moreover, the emission intensity at 511 nm was linearly proportional (correlation coefficient, R^2^ > 0.99) to the concentration of Fe^3+^ over the range of 0–30 μM, with a limit of detection of 1.93 µM (Fig. S3). These observations revealed that **FH** is suitable for use as a sensor for the quantitative measurement of Fe^3+^. To investigate the binding stoichiometry between **FH** and Fe^3+^, a Job’s plot experiment was carried out by keeping the total concentration of **FH** and Fe^3+^ ions at 20 μM and changing the molar ratio of Fe^3+^ from 0 to 1. As shown in Fig. S4 the result indicates a maximum molar fraction of 0.7, indicating the formation of 1:2 complex of **FH** and Fe^3+^. This agrees with complexes previously reported^83,84^. On the basis of changes in emission intensity at 511 nm, the stoichiometric ratio and apparent binding constant of **FH** with Fe^3+^ was determined using Benesi–Hildebrand (B-H) linear regression analysis. From the B − H plot, a 1:2 stoichiometry between **FH** with Fe^3+^ was confirmed with an association constant of 5.25 × 10^3^ M^−1^ (Fig. S5).

### Competition experiment using fluorescence spectroscopy

**Figure 5:**
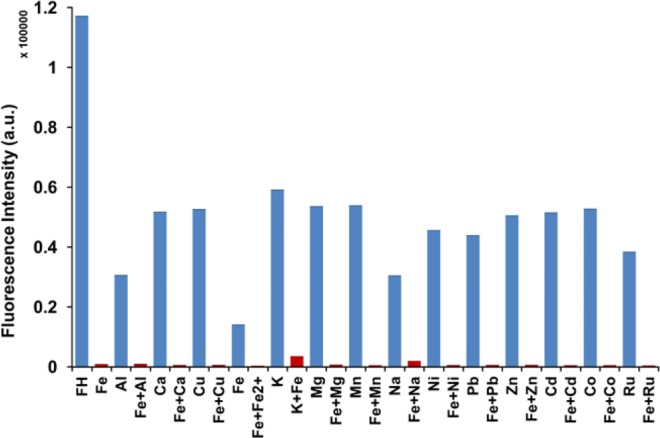
Competitive experiments in the **FH** + Fe^3+^ system with potential interfering metal ions. **FH** (0.5 μM), Fe^3+^ (50 μM), and other metals (50 μM). Excited at 374 nm and emission measured at 511 nm.

To further investigate the practical applicability of **FH** as a selective sensor for Fe^3+^, a competition experiment was carried out for **FH** in the presence of Fe^3+^ mixed with other metal ions (Na^+^, K^+^ Mg^2+^, Ca^2+^, Fe^2+^, Mn^2+^, Al^3+^, Ni^2+^, Cu^2+^, Zn^2+^, Co^2+^, Pb^2+^, Ru^3+^). Interestingly, the fluorescence emission intensity was quenched in every case after mixing Fe^3+^ with each of the candidate metal ions (Fig. 5). Thus, **FH** shows great promise as a highly selective and sensitive fluorescence turn-off sensor for the detection of Fe^3+^ even in the presence of other analogous ions (in particular, Fe^2+^ and Ru^3+^). Furthermore, based on the general trend in Fig. 5, it is apparent that 3+ cations tend to exhibit stronger binding that effects fluorescence quenching of **FH**. This may be due to stabilization of the binding with an anion (NO^3−^); 2 bonds at **FH** and one bond with anion. Consider, for example Al^3+^, where the cation can bind in a similar way. This tridentate binding is certainly more stable than the other 2+ cations with bidentate binding. It is also apparent that Fe^3+^ shows better binding with **FH** than Fe^2+^ which can be attributed to the cationic radii, since Fe^3+^ is much smaller than Fe^2+^ about half the size of the Fe^3+^ radius^85^. When considering 1+ cations it is interesting that Na^+^ also quenches **FH** but with K^+^ to a lesser extent. This is probably related to the single bond with **FH** that is not very stable. Moreover, Na^+^ has better electronegativity compared to K^+^, which one expects promotes better binding with **FH**.

### Proposed sensing mechanism

**Figure 6:**
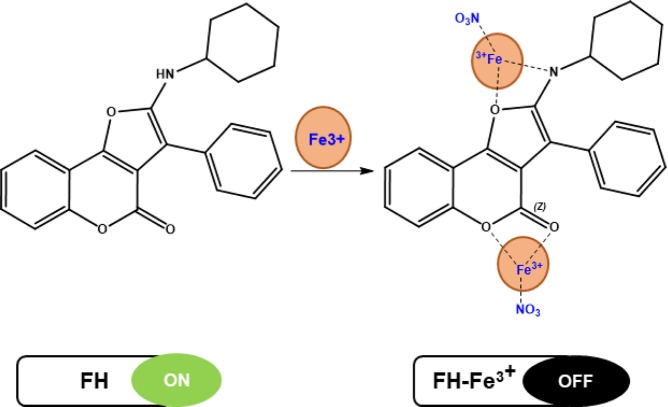
Proposed binding mode of **FH** with Fe^3+^.

To study the reasonable binding mode of **FH** and Fe^3+^, mass spectrometry analysis has been carried out and supports the formation of a 1:2 **FH**-Fe^3+^complex. As illustrated in Fig. S6, **FH** exhibits an intense protonated peak at *m*/*z* 360.21, while in the presence of Fe^3+^, a peak at *m*/*z* 595.55 is observed, which is attributed to the formation of a protonated FH:(Fe^3+^NO_3_)_2_ complex. For the mentioned results above, as well as the Job’s plot (Fig. S4), we suspect that the sensing mechanism for the 1:2 binding modes of the **FH**-Fe^3+^complex is as suggested in Fig. 6. IR spectroscopy was used to elucidate the coordination mode between **FH** and Fe^3+^ (Fig. S7), shows the FTIR spectra of **FH** before and after the addition of Fe^3+^. A shift in the characteristic absorption band in the FTIR spectra confirmed the coordination behavior for **FH**-Fe^3+^. Upon the introduction of Fe^3+^, an extremely broad peak appeared between 3665 and 3125 cm^−1^, which is attributed to the involvement of nitrogen from the primary amine (NH) and oxygen from furan in the binding of Fe^3+^. Furthermore, the stretching vibration frequency of the pyrone carbonyl (C=O) at 1720 cm^−1^ is shifted to 1605 cm^−1^.

### Fluorescence and UV–vis titration studies of FH with other metal ions (in water/methanol (9:1, v/v))

**Figure 7:**
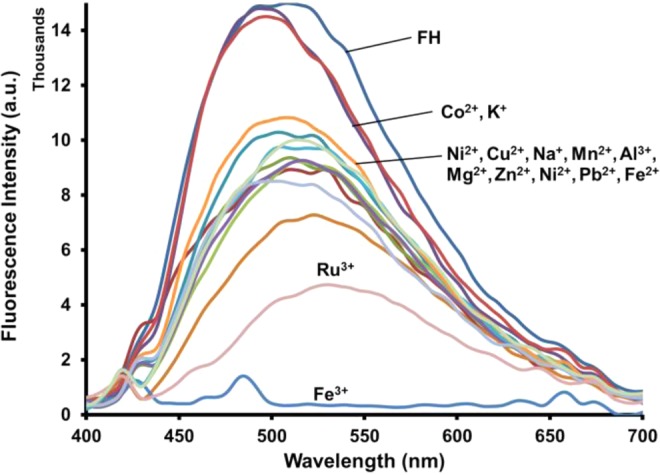
Fluorescence spectra of **FH** (5 μM) in the presence of different metal ions (10 equiv.) in water/methanol (9:1, v/v).

**Figure 8:**
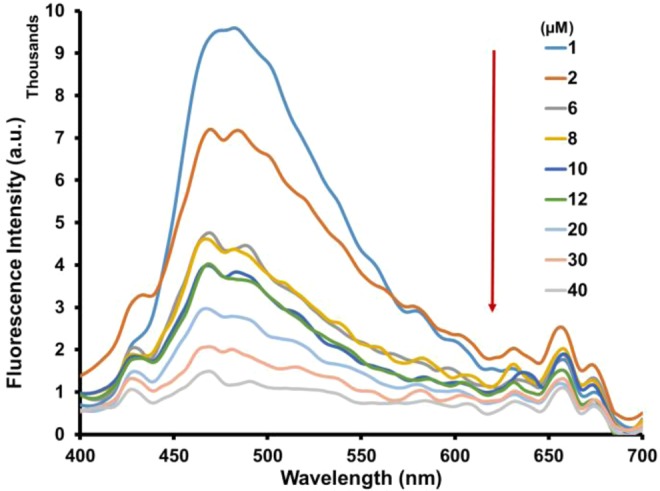
Fluorescence emission spectra of **FH** (5 μM) titrated with Fe^3+^ (0.2–8 equiv.) in water/methanol (9:1, v/v).

**Figure 9:**
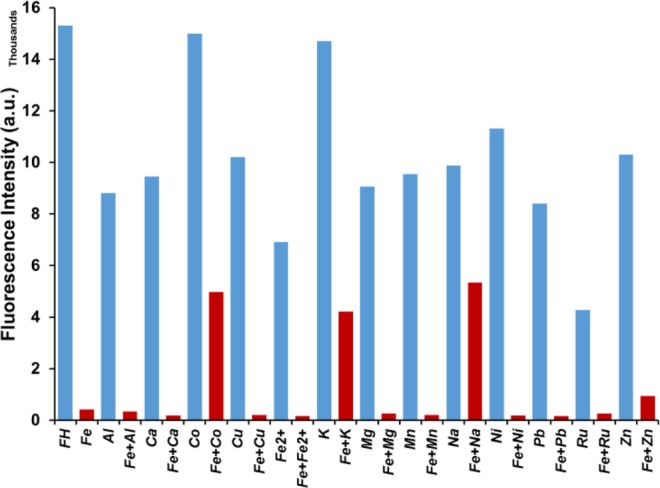
Competitive experiments in the **FH** + Fe^3+^ system with interfering metal ions. **FH** (5 μM), Fe^3+^ (50 μM) and other metals (50 μM) in water/methanol (9:1, v/v). Excited at 374 nm and emission measured at 511 nm.

Fluorescence quenching in protic solvents is a common problem with previously reported fluorescence sensors^86^, In order to confirm **FH** is not susceptible to this issue and to demonstrate a real-world sample application, the photophysical properties of sensor **FH** were examined in a predominantly aqueous environment, water/methanol (9:1, v/v) at 5 µM. This composition of 9:1 v/v water/methanol was at the maximum solubility of FH in water. Changes to the fluorescence properties of **FH** caused by various metal ions are shown in Fig. 7. The result shows Fe^3+^ also produces significant quenching in the fluorescent emission of **FH**. The other tested metals only show relatively insignificant changes, except Co^2+^, Na^+^ and K^+^. So, it can be concluded that **FH** also has high selectivity for recognition of Fe^3+^ in a predominantly aqueous solution. The fluorescence spectra of **FH** (5 µM) in water/methanol (9:1, v/v), in the presence of various concentrations of Fe^3+^ ion (0.2–8 equiv.), are shown in Fig. 8, which shows quenching in the fluorescent emission of **FH** when the concentration of Fe^3+^ is increased. A Job’s plot of **FH** with Fe^3+^ also indicates the formation of a 1:2 complex (Fig. S8). A competitive assay (Fig. 9) confirms that **FH** can still detect Fe^3+^ even in the presence of other heavy metals. Thus, in a predominantly aqueous solution, **FH** exhibits high selectivity for Fe^3+^ over the other tested metal ions except Co^2+^, Na^+^ and K^+^.

### Determination of Fe^3+^ in real water samples

To investigate the applicability of the **FH** sensor in realistic environmental samples, recovery studies were carried out in mineral drinking water and tap water samples doped with Fe^3+^, using fluorescence emission spectroscopy. Testing on these water samples was performed without any sample pre-treatment except for the addition of **FH**, Fe^3+^ and allowing 1 minute for mixing. From Table 2, we can see that the recoveries of Fe^3+^ were from 91.5% to 125%. These data indicate that **FH** as a sensor has significant potential for the practical detection of Fe^3+^ in various aqueous samples where other potentially competing species are present.

**Table 2:** Analytical results of **FH**-Fe^3+^ in water samples.

Water samples	Added (µM)	Found (µM)	Recovery (%)	RSD (%)
Mineral water	2.0	2.1	105	0.71
	10.0	10.0	100	5.71
	20.0	19.3	96.5	9.60
Tap water	2.0	2.5	125	0.5
	10.0	9.8	98	6.9
	20.0	18.3	91.5	4.2

## Conclusion

In summary, we have successfully synthesized and for the first time, characterized, the fluorescence properties of furocoumarin derivatives (**FH**, **FCl** and **FNO**_**2**_). These were synthesized by mixing 4-hydrocoumarin, benzaldehyde derivatives, and cyclohexyl isocyanide under reflux conditions within 24 h using singlestep high yielding chemistry (82–92% yield). All compounds are purified from recrystallisation preventing the need for time consuming column chromatography and showing that this chemistry is amenable to automated high throughput synthesis and screening technologies. Both **FH** and **FCl** produce strong fluorescence intensity whilst **FNO**_**2**_ does not, as a result of strong electron withdrawing from –NO_2_ causing fluorescence quenching of furocoumarin. Furthermore, the fluorescence study has led us towards a successful demonstration of a novel coumarin-based fluorescent (**FH**) ratiometric chemosensor, with an LMCT mechanism attributed to the recognition of Fe^3+^ in methanol and also in water/methanol (9:1, v/v). **FH** formed 1:2 complexes with Fe^3+^ and exhibited a fluorescence turn-off response to Fe^3+^. Extensive competitive selectivity experiments in methanol have been performed for Na^+^, K^+^, Mg^2+^, Ca^2+^, Mn^2+^, Fe^2+^, Al^3+^, Ni^2+^, Cu^2+^, Zn^2+^, Co^2+^, Pb^2+^ and Ru^3+^ demonstrating that **FH** has higher selectivity towards Fe^3+^ (fluorescence turn-off) than other analogous ions and other previously reported Fe^3+^ sensors (to the best of our knowledge). In an aqueous environment the probe selectivity reduces but the “turn off” effect is still operational confirming water does not fully quench fluorescence. The potential of this sensor has been further highlighted by testing with untreated mineral and tap water samples. This result sets the foundation for a second generation of sensors with improved sensing properties and water solubilizing groups with the real potential of developing a fully aqueous furocoumarin based sensor, which is the subject of future work.

## Materials and Methods

### Materials

All reagents were purchased from commercial suppliers and used without further purification. The salts used in stock solutions of metal ions were Al(NO_3_)_3_.9H_2_O, CaCl_2_, CoCl_2_.6H_2_O, Cu(NO_3_)_2_.4H_2_O, FeCl_2_.4H_2_O, Fe(NO_3_)_3_.9H_2_O, KOH, MgCl_2,_ MnCl_2_, NaOH, NiCl_2_.6H_2_O, Pb(NO_3_)_2_, RuCl_3_ · H_2_O, Zn(NO_3_)_2_ · 6H_2_O.

### Instrumentation

^1^H NMR (400 MHz) spectra were acquired on a Bruker AVANCE 400 MHz NMR Spectrometer using TMS (tetramethylsilane) as internal standard. All stock solutions of the samples for both UV-Vis and Fluorescence studies were prepared at 0.1 mM in different solvents (ethanol, chloroform and ethyl acetate) and diluted in 10 mL with appropriate concentrations. UV-vis absorption and fluorescence spectra of the furocoumarin derivatives (in solution) were recorded on a CARY 60 UV-Vis spectrophotometer and CARY Eclipse Fluorescence Spectrometer, respectively. Excitation and emission monochromator band pass were kept at 5 nm using a quartz cell cuvette (1 × 1 cm). The absolute quantum yields were calculated using quinine sulfate in 0.1 M H_2_SO_4_ as a standard. Fluorescence lifetime measurements were performed with the use of an FLS 1000 Spectrometer (Edinburgh Instruments, Livingston, UK) at room temperature. In these experiments the fluorescence lifetimes of the furocoumarin derivatives in methanol were measured using the photon counting technique (requiring at least 10,000 photons per second to be counted because the signal-to noise ratio becomes unsatisfactory at lower count rates^87^) with an excitation wavelength set to 374 nm in all the cases. UV-vis absorption and fluorescence spectra of **FH** and all metal ions were performed with the use of a Cary 5000 UV-Vis-NIR Spectrophotometer (Agilent Technologies) and FLS 1000 Spectrometer (Edinburgh Instruments), respectively. Paper spray ionization mass spectrometry (PSI-MS)^88–90^ was performed on a Waters Xevo TQ-MS (Waters, Wilmslow, UK).

### Synthesis of furo [3,2-c] coumarin derivatives

**Figure 10:**
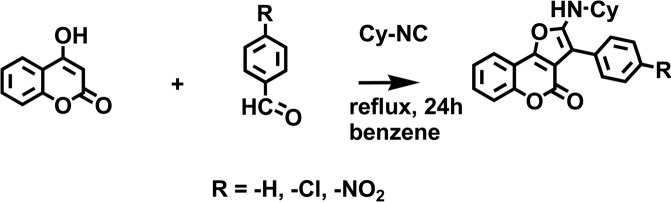
Synthesis of furo[3,2-c]coumarin derivatives.

Equimolar amounts of 4-hydroxycoumarin and benzaldehyde derivatives were dissolved in benzene (0.2 M) and heated under reflux (Fig. 10). After 30 minutes, cyclohexyl isocyanide (1 eq.) was added to the reaction mixture and further refluxed for 24 h. The pure compound was obtained by recrystallization from diethyl ether to produce up to 85% yield. These compounds have been reported and the characterization data agree with previous studies^18,19^.

2-(Cyclohexylamino)-3-phenyl-4H-furo[3,2-c]chromen-4-one. **FH**, 92% yield, light yellow powder, m.p. = 120–122 °C, FTIR = 3250 (NH), 2925–2850 (cyclohexane), 1720 (C=O of pyrone), 1570 (C=C of pyrone), ^1^H NMR = 1.18–2.08(m, 10H), 3.55–3.58 (m, 1H), 4.29 (d, J = 8.32 Hz 1H), 7.27–7.31 (m, 2H), 7.39 (d, J = 4 Hz, 1H), 7.43 (t, J = 8H, 3H), 7.52 (d, J = 8 Hz, 2H), 7.77 (d, J = 8 Hz, 1H), ^1^H NMR spectrum of **FH** as shown in Fig. S9. UV-Vis = 375 nm (in ethanol).

2-(Cyclohexylamino)-3-(4-chlorophenyl)-4H-furo[3,2-c]chromen-4-one. **FCl**, 90% yield, bright crystalline yellow, m.p. = 110–112 °C, FTIR = 3289 (NH), 2930–2857 (cyclohexane), 1707 (C=O of pyrone),1593 (C = C of pyrone), ^1^H NMR = 1.16–2.07 (m, 10H), 3.57 (br, 1H), 4.21 (s, 1H), 7.33–7.28 (m, 1H), 7.41–7.39 (m, 4H), 7.47 (d, J = 6.4 Hz, 2H), 7.77 (d, J = 7.6 Hz, 1H), ^1^H NMR spectrum of **FCl** as shown in Fig. S10. UV-Vis = 375 nm (in ethanol).

2-(Cyclohexylamino)-3-(4-nitrophenyl)-4H-furo[3,2-c]chromen-4-one. **FNO**_**2**_, 85% yield, reddish orange powder, m.p. = 145–147 °C, 3389 (NH), 2929–2851 (cyclohexane), 1736 (C=O of pyrone), 1574 (C=C of pyrone), ^1^H NMR = 1.19–2.11 (m, 10H), 3.67 (m, 1H), 4.60 (d, J = 7.96 Hz 1H), 7.34 (t, J = 6.80 Hz, 1H), 7.45–7.40 (m, 2H) 7.69 (d, J = 8.72 Hz, 2H), 7.77 (d, J = 7.64 Hz, 1H), 8.22(d, J = 8.64 Hz, 2H), ^1^H NMR spectrum of **FNO**_**2**_ as shown in Fig. S11. UV-Vis = 380 nm (in ethanol).

### Fluorescence spectral responses of FH to metal ions

The analysis was conducted for two different solvent systems: pure methanol and a water/methanol mixture (9:1, v/v). All stock solutions of the furocoumarin (FC) and various metal ions (Mg^2+^, Ca^2+^, Mn^2+^, Fe^2+^, Fe^3+^, Al^3+^, Ni^2+^, Cu^2+^, Zn^2+^, Co^2+^, Pb^2+^ and Ru^3+^) were analyzed at a concentration of 0.001 M, except Na^+^ and K^+^ at 0.2 M in methanol. Then, each of the metal ions were diluted to 50 μM, while **FH** was diluted to 0.5 μM in methanol. For the water/methanol solvent system, **FH** was diluted to 5 μM.

For testing, **FH** was mixed with each of the metal ions for up to 1 minute (by stirring until no layers could be visually observed) after which UV-Vis and fluorescence analysis were carried out. The fluorescence emission spectra were recorded from 430 to 700 nm with an excitation wavelength at 374 nm. Both excitation and emission slit widths were set at 1 nm. For the competing analysis, the fluorescence changes of **FH** in methanol were measured by the treatment of 50 μM Fe^3+^ ion in the presence of 50 μM other interfering metal ions. All of the background metal ions tested showed no interference with the detection of Fe^3+^ by competitive experiment.

## Supplementary information

Supplementary information.
