# Supplementary material for: Furo[3,2-c]coumarin-derived Fe3+ Selective Fluorescence Sensor: Synthesis, Fluorescence Study and Application to Water Analysis
Source: Sci Rep. 2020 May 4;10:7421. doi: 10.1038/s41598-020-63262-7 (PMC7198544; doi:10.1038/s41598-020-63262-7)
Supplement: Supplementary file 1 — Supplementary information. [file 41598_2020_63262_MOESM1_ESM.docx]

**Supporting Information**

**Furo[3,2-c]coumarin-derived Fe^3+^ Selective Fluorescence Sensor: Synthesis, Fluorescence Study and Application to Water Analysis**

Norfatirah Muhamad Sarih^1,2^, Alexander Ciupa^3^, Stephen Moss^4^, Peter Myers^4^, Anna Grace Slater^3,4^, Zanariah Abdullah^2,^ Hairul Anuar Tajuddin^2,+^ and Simon Maher^1,^*

^1^ Department of Electrical Engineering and Electronics, University of Liverpool, Brownlow Hill, Liverpool, L69 GJ, UK; *email: s.maher@liverpool.ac.uk

^2^ Department of Chemistry, Faculty of Science, University of Malaya, 50603 Kuala Lumpur, Malaysia; ^+^email: hairul@um.edu.my

^3^ Materials Innovation Factory, University of Liverpool, 51 Oxford St, Liverpool, L7 3NY, UK.

^4^ Department of Chemistry, University of Liverpool, Crown St, Liverpool, L69 7ZD, UK.

For correspondence, email: s.maher@liverpool.ac.uk


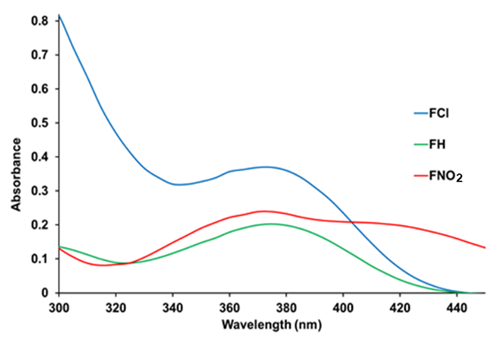


**Figure S1**. UV-Vis Spectra of Furocoumarin derivatives (**FC**, **FH**, **FNO_2_**) in ethanol.


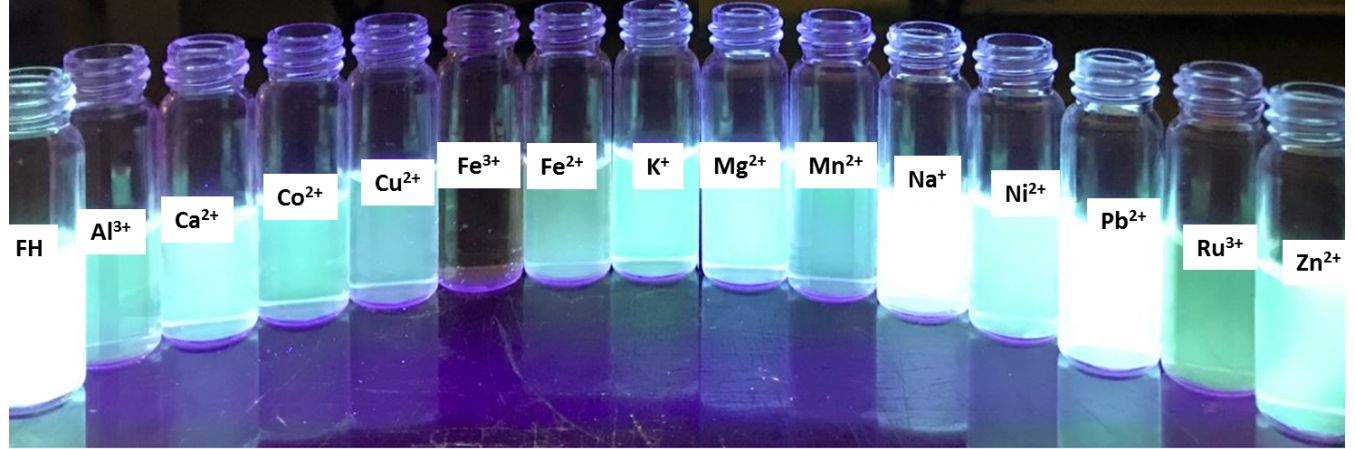


**Figure S2**. Photograph of **FH** on its own and mixed with various metal ions (as labelled) under UV light illumination (380 nm).

**
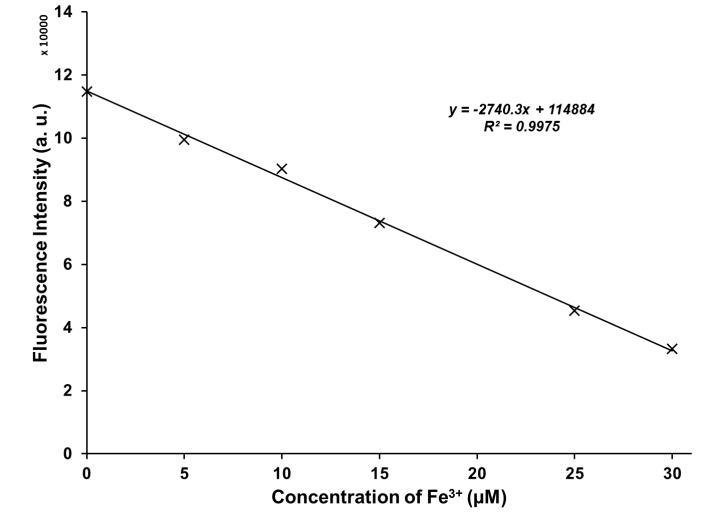
**

**Figure S3**. Calibration curve based on FH titration with Fe^3+^ in methanol.


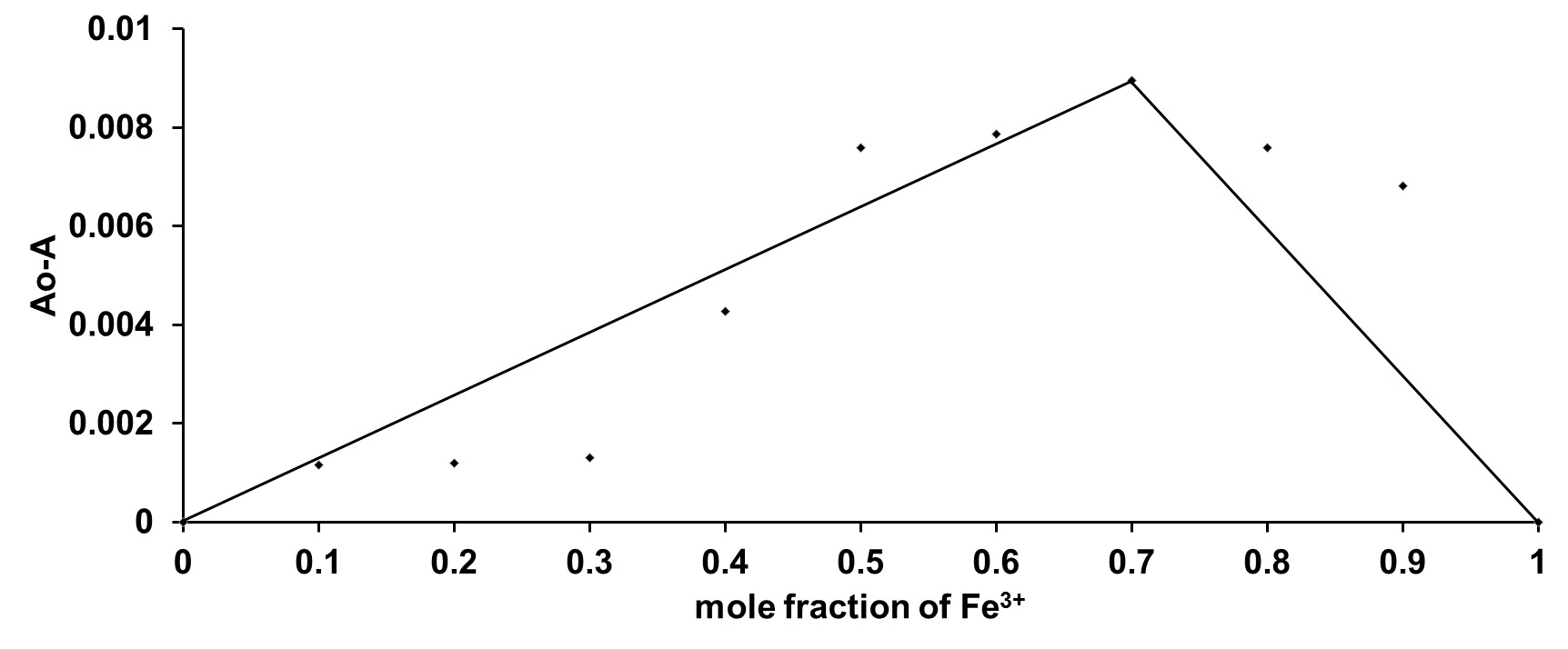


**Figure S4**. Job’s plot for determining the stoichiometry for FH and Fe^3+^ in methanol. Total concentration= 2x10^-5^M.


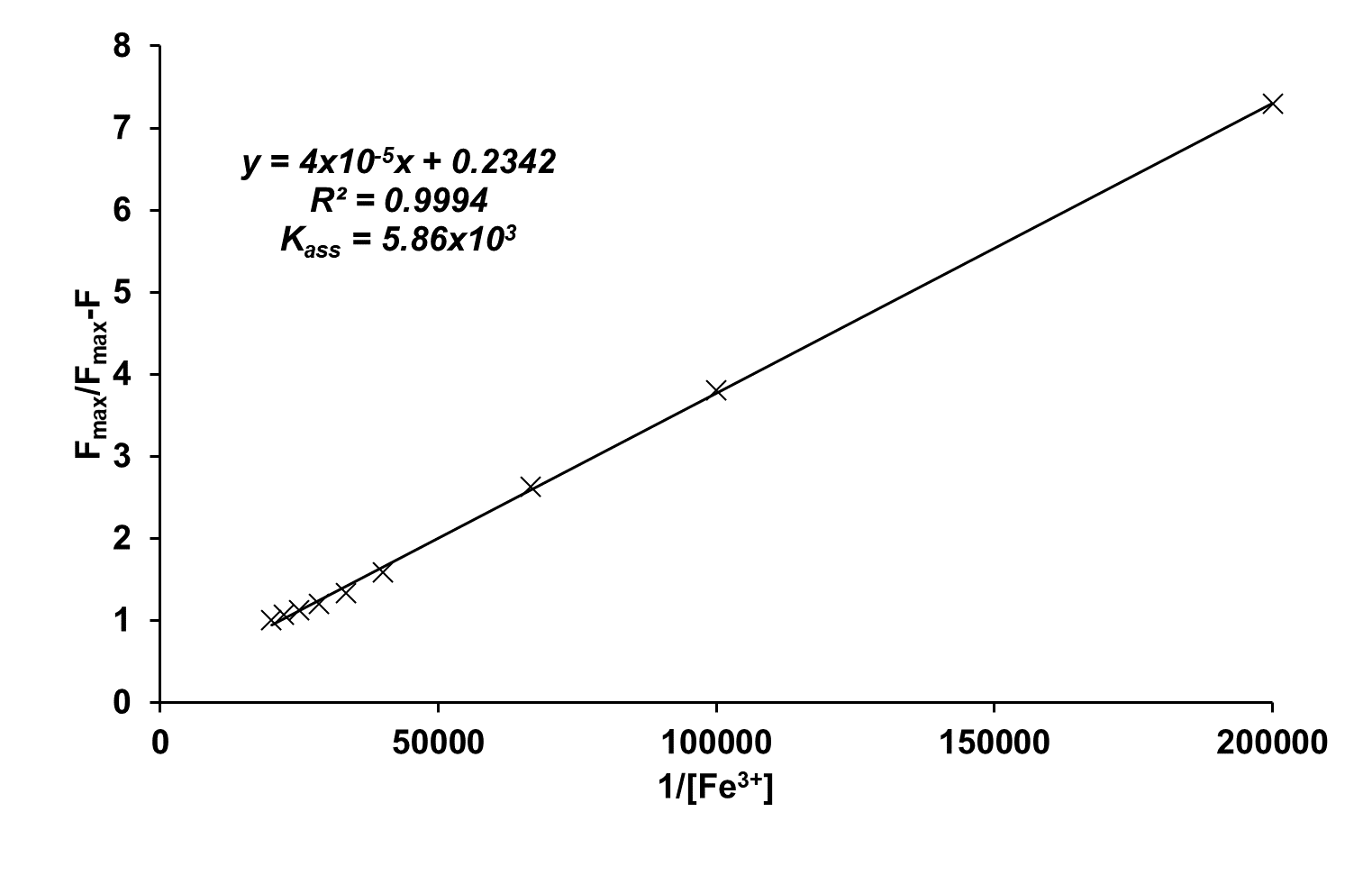


**Figure S5**. Benesi–Hildebrand plot of **FH** sensor with Fe^3+^.


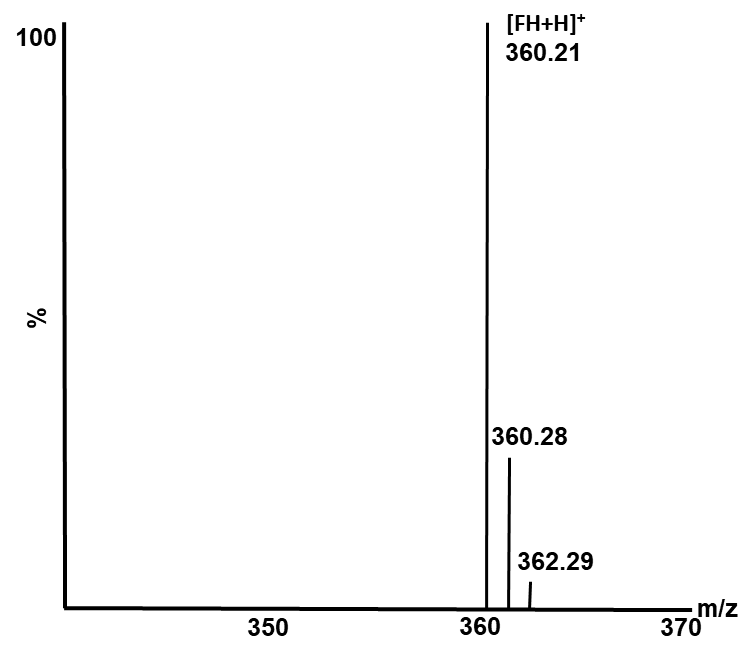


(a)

**
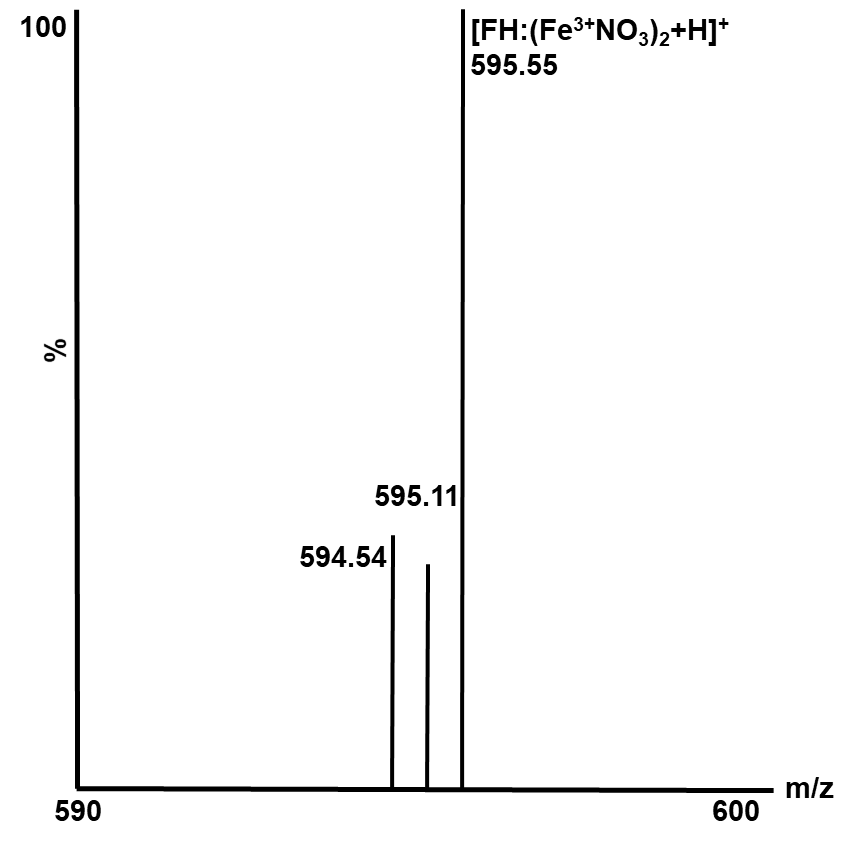
**

(b)

**Figure S6.**  Mass spectra of (a) **FH** in acetonitrile and (b) **FH** mixed with Iron Nitrite.

**
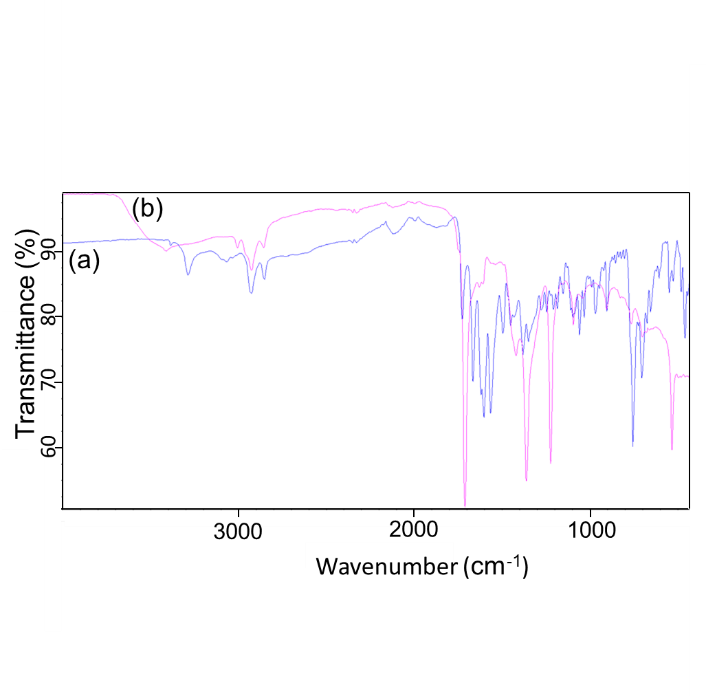
**

**Figure S7**. IR spectra of **FH** (a) before and (b) after the addition of Fe^3+^.

**
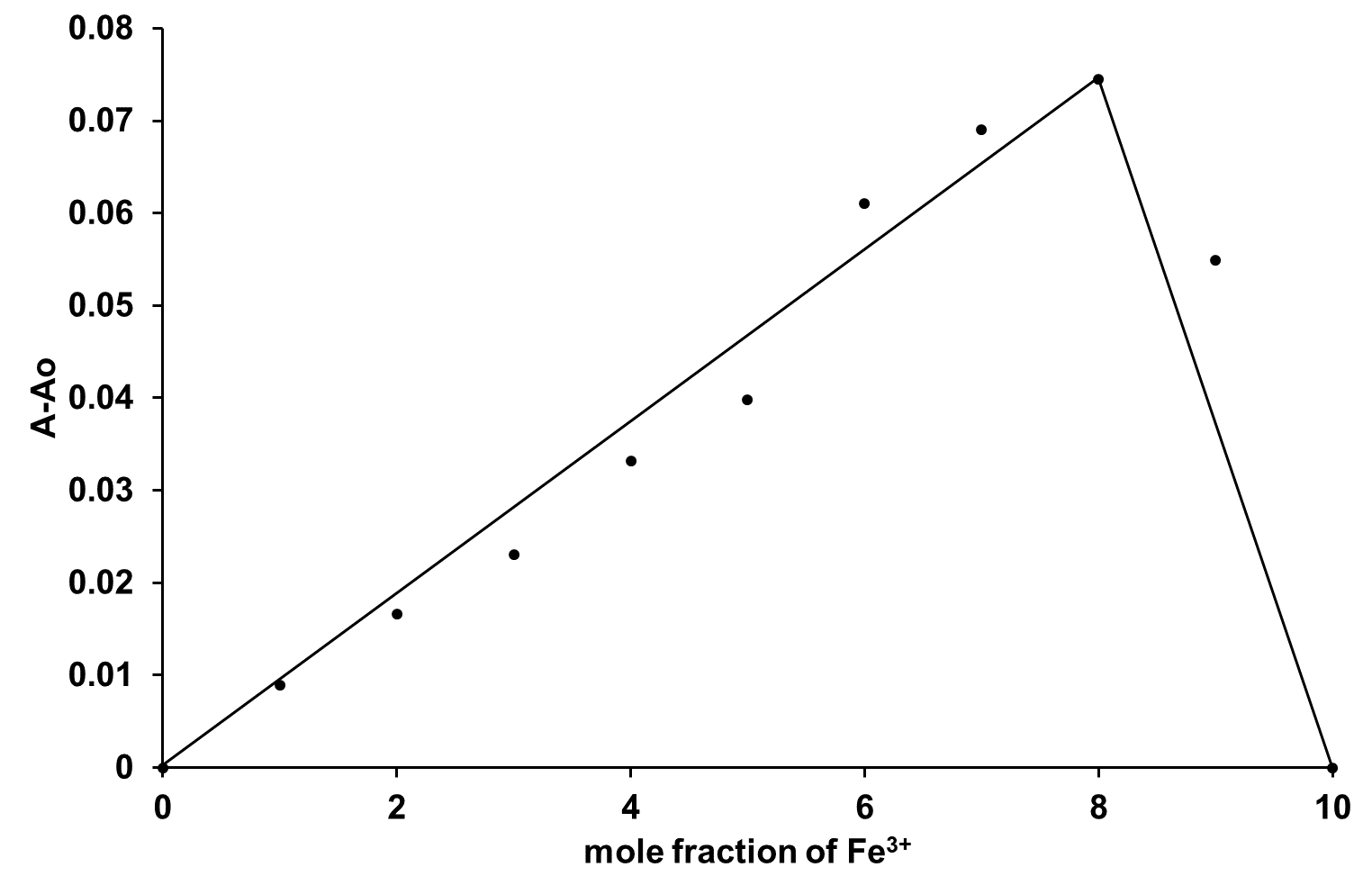
**

**Figure S8.** Job’s plot for determining the stoichiometry for **FH** and Fe^3+^ in water/methanol (9:1, v/v). Total concentration = 2x10^-5^M.

**Application with Real Water Samples**

Mineral water samples were directly purchased from a local supermarket (Brand: Volvic, Source: Clairvic Spring, Auvergne Regional Park, at the North of the Puy de Dôme in France) and tap water samples were obtained from our laboratory. The fluorescence intensities of samples were analysed after being spiked with a standard solution of Fe^3+^.


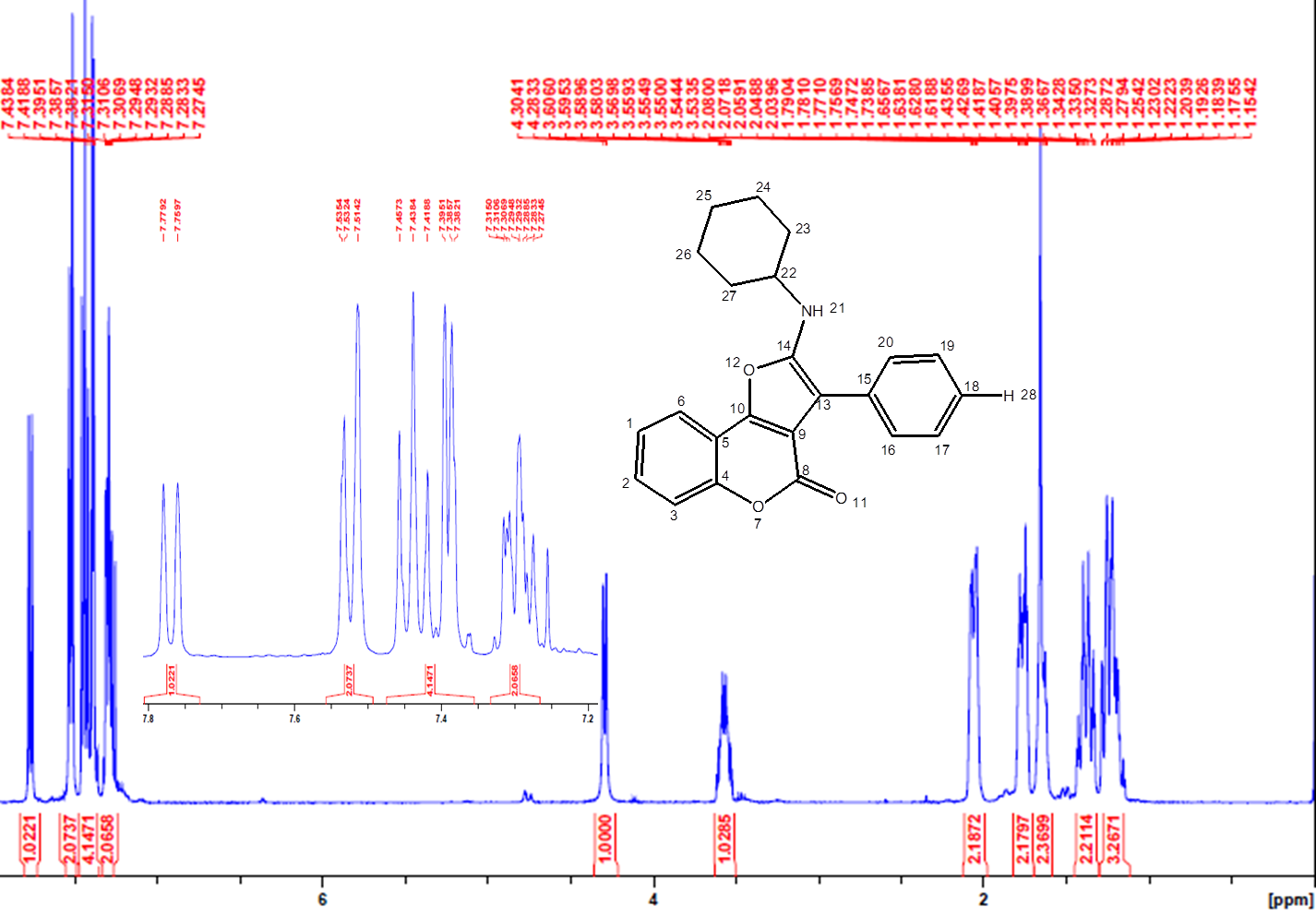


**Figure S9**. ^1^H NMR spectrum of **FH.**


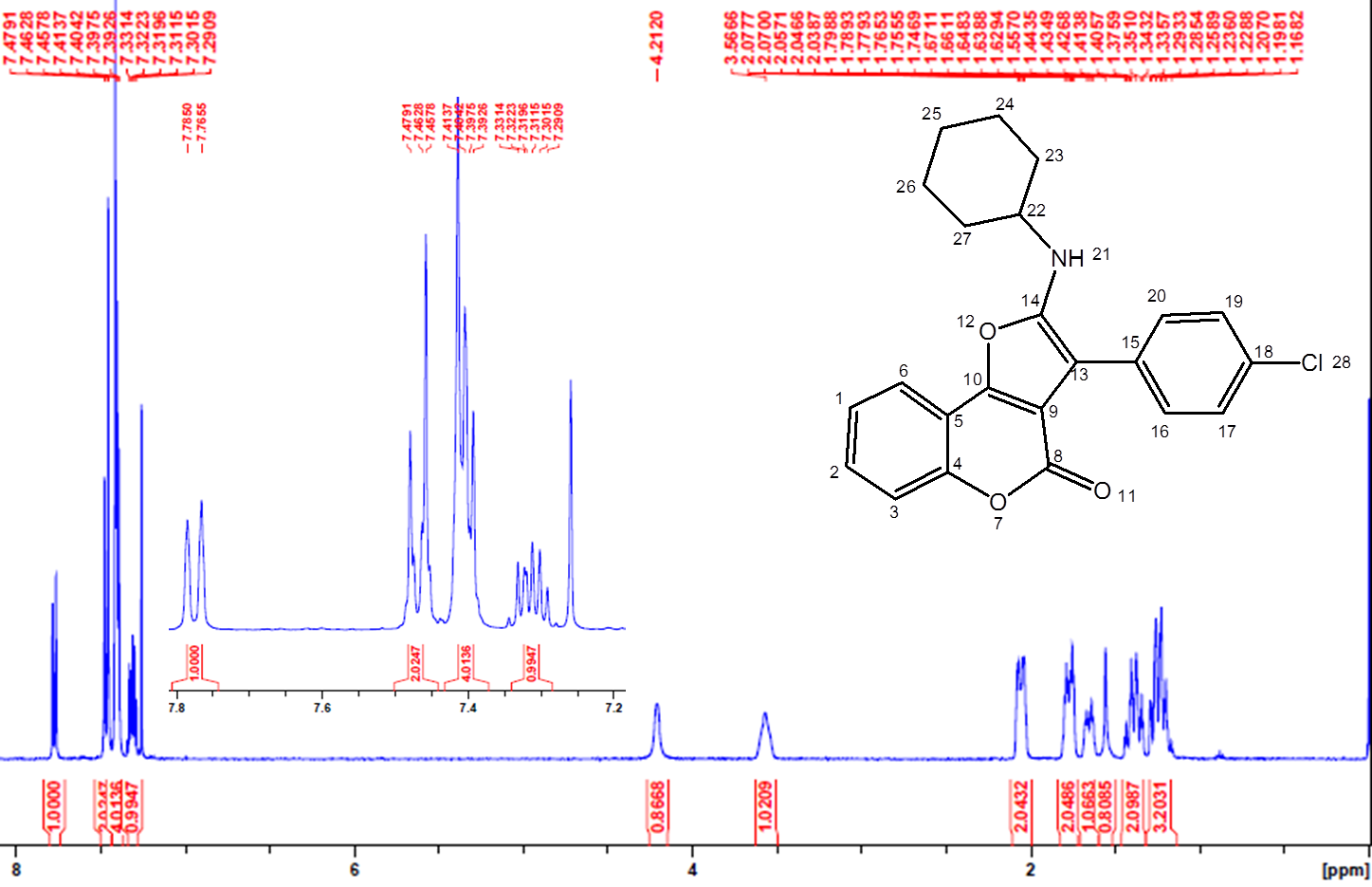


**Figure S10.** ^1^H NMR spectrum of **FCl.**


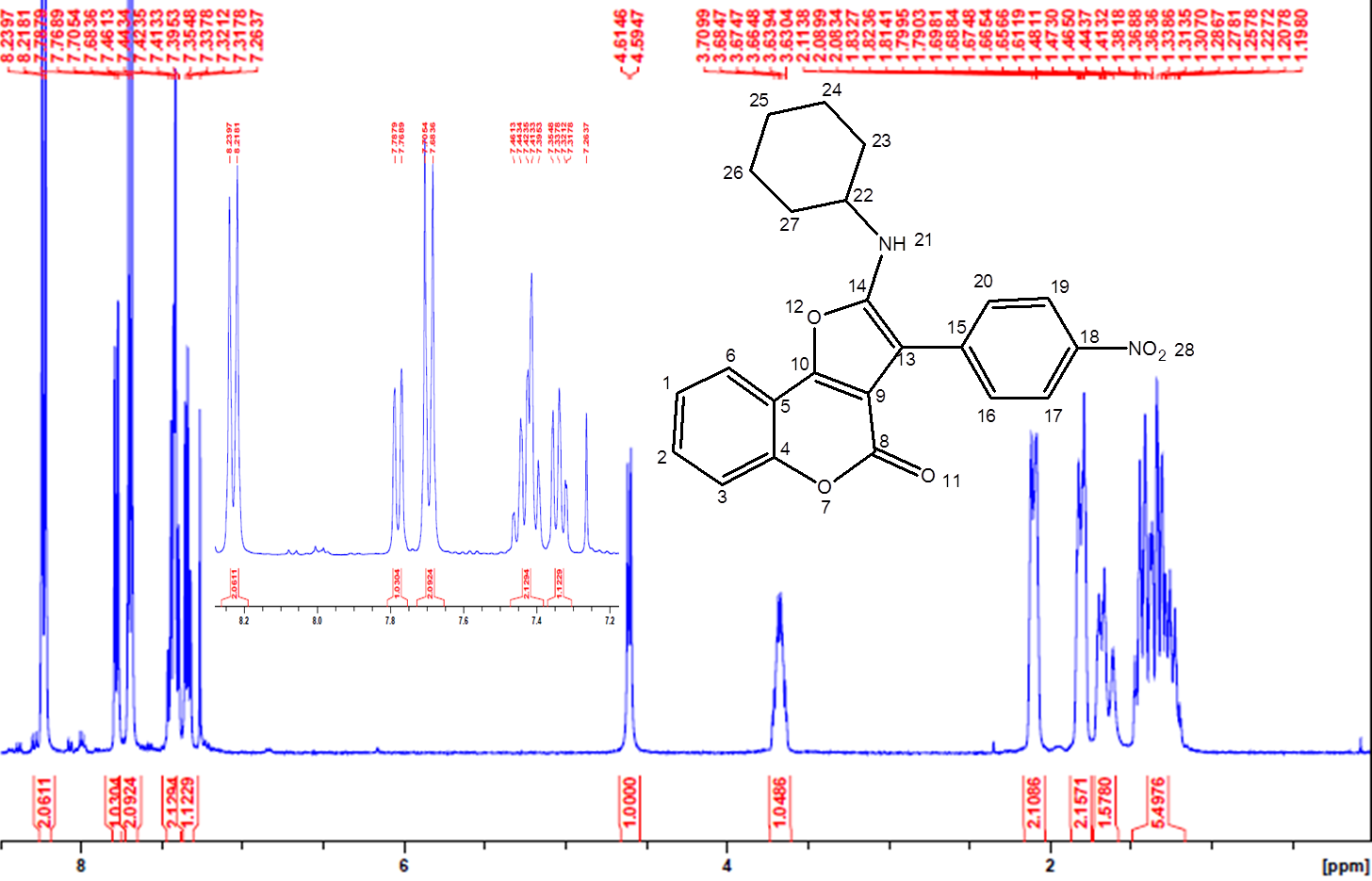


**Figure S11.** ^1^H NMR spectrum of **FNO_2._**
